# Early Mobilization Interventions in the Intensive Care Unit: Ongoing and Unpublished Randomized Trials

**DOI:** 10.1155/2020/3281394

**Published:** 2020-01-21

**Authors:** Janane Maheswaran, Jake Fromowitz, Michael Goldfarb

**Affiliations:** ^1^Department of Medicine, Jewish General Hospital, McGill University, Montreal, Quebec, Canada; ^2^Nova Southeastern University, Fort Lauderdale, FL, USA; ^3^Division of Cardiology, Jewish General Hospital, McGill University, Montreal, Quebec, Canada

## Abstract

**Background:**

Critical care societies recommend early mobilization (EM) as standard practice in the intensive care unit (ICU) setting. However, there is limited randomized controlled trial (RCT) evidence supporting EM's effectiveness. Our objective was to identify ongoing or completed RCTs assessing EM's effectiveness in the ICU.

**Method:**

We searched ClinicalTrials.gov and the Australian New Zealand Clinical Trials Registry for ongoing or completed but not published RCTs in an ICU setting with objective outcome measures.

**Results:**

There were 14 RCTs included in the analysis. All studies were in the general or mixed ICU setting (*N*=14). Half of the studies (*N*=7) were small RCTs (<100 projected participants) and half (*N*=7) were medium-sized RCTs (100–999 participants). Inclusion criteria included mechanical ventilation use or expected use (*N*=13) and prehospital functional status (*N*=7). Primary EM interventions were standard physiotherapist-based activities (*N*=4), cycling (*N*=9), and electrical muscle stimulation (*N*=1). Only one study involved nurse-led EM. The most common assessment tool was the 6-minute walk test (*N*=6). Primary outcome measures were physiological (*N*=3), clinical (*N*=3), patient-centered (*N*=7), and healthcare resource use (*N*=1). Most studies (*N*=8) involved post-ICU follow-up measures up to 1-year posthospitalization. There were no studies targeting older adults or people with acute cardiac disease.

**Conclusion:**

Identified studies will further the evidence base for EM's effectiveness. There is a need for studies looking at specific patient populations that may benefit from EM, such as older adults and cardiac patients, as well as for novel EM delivery strategies, such as nurse-led EM.

## 1. Background and Rationale

Early mobilization (EM) involves progressive mobilization activities that start upon hemodynamic and respiratory stabilization, typically within 24–48 hours of intensive care unit (ICU) admission [1]. The goal of EM is to prevent loss of muscle function, promote mobility recovery, and maintain or help patients regain prehospital functional capabilities. There is observational evidence that EM in the ICU is associated with improvements in muscle strength and physical function, decreased rates of delirium, and reduced healthcare resource utilization (shorter ICU and hospital stays and reduced hospital readmission rate) [2–6]. The safety and feasibility of EM in critically ill patients has also been established [7]. As a result, critical care professional societies recommend EM as part of routine ICU care [8, 9].

**Figure 1:**
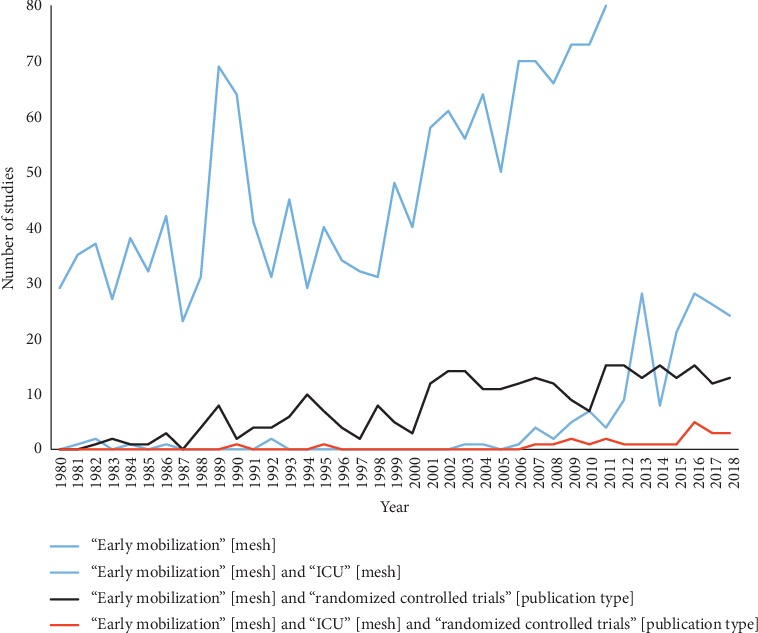
PubMed entries for early mobilization by year of publication.

Yet despite the evidence for safety and feasibility, there is limited randomized evidence supporting the effectiveness of EM in the ICU setting [10]. A recent Cochrane review of randomized controlled trials (RCTs) of EM interventions in the ICU found only 4 studies with 690 patients and concluded that there was insufficient randomized evidence for EM's effectiveness [10]. While there has been a considerable number of EM observational studies, there have been relatively few published RCTs (Figure 1). As a result, there is limited good quality evidence for the ability of EM to maintain or improve physical function at discharge and patient-centred outcomes following hospital discharge. A lack of strong evidence for EM's effectiveness is a noted barrier to the implementation of EM [11].

Given the dissonance between EM's current evidence base and critical care societal recommendations for EM, there is a need to characterize upcoming studies that could provide stronger evidence for EM's benefits. Thus, the objective of this review was to identify ongoing or completed but not yet published EM RCTs in the ICU setting. We also identified important areas of EM research that currently lack data and that could be addressed in future research design.

## 2. Methods

We searched ClinicalTrials.gov and the Australian New Zealand Clinical Trials Registry (ANZCTR), two web-based databases that list ongoing and completed RCTs. ClinicalTrials.gov is operated by the United States National Library of Medicine and contains more than 130,000 trials from more than 170 countries worldwide. The ANZCTR is an Australian government-funded registry of clinical trials that includes studies primarily, but not exclusively, from Australia and New Zealand. The ANZCTR was included in the search since a preliminary literature search and prior systematic reviews identified a research group in Australia and New Zealand that was active in EM research. For both of these databases, we used the following search query: (early mobilization OR early mobilization OR early mobility OR early rehab OR early rehabilitation) AND (randomized). Studies were included if they were (1) randomized controlled trials, (2) ongoing or completed but not yet published, (3) in the ICU setting, and (4) had an objective outcome measure. Studies were excluded if they were observational studies, in a pediatric population, terminated prematurely, involved cohorts of patients post-orthopedic surgery, poststroke, and posttraumatic brain injury, as these patients possess specific deficits with specialized needs for mobilization and physical rehabilitation. Studies were also excluded if they were completed but unpublished for 5 or more years, as the likelihood of eventual publication was low. Two independent reviewers (J. M. and J. F.) reviewed all the studies that met inclusion criteria for suitability into the analysis; disagreements were resolved by a third reviewer (M. G.). To confirm unpublished status, PubMed and Web of Knowledge were queried for each eligible trial. If no published study was found, attempts to contact authors by e-mail were made. The search was performed on December 19, 2018. Trial characteristics were retrieved from the study listings and from trial methodology papers when available. Attempts to contact authors by e-mail were made for missing or unavailable data. Projected study size was defined as small (<100 participants), medium (100–999 participants), and large (≥1000 participants).

## 3. Results

**Figure 2:**
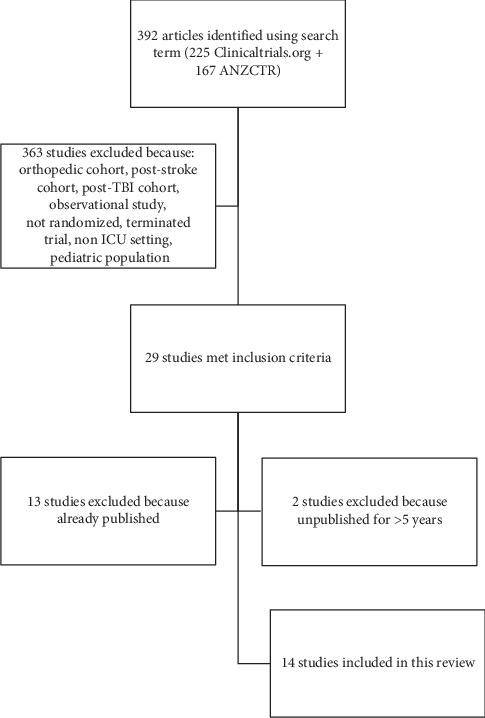
Search strategy flow diagram. Abbreviations: ANZCTR, Australian New Zealand Clinical Trials Registry; ICU, intensive care unit; TBI, traumatic brain injury.

There were 392 studies identified by the search, of which 14 were included in the analysis (Figure 2; Table 1). All studies (*N*=14) involved the general ICU setting. The projected study size was small (*N*=7) or medium (*N*=7) RCTs, with study populations ranging in size from 28 to 772 participants. Nearly all studies (*N*=13) involved mechanical ventilation (MV) as part of the inclusion criteria. Other inclusion criteria included prehospital functional status (*N*=7) and expected prolonged MV (*N*=7). Exclusion criteria involved moribund patients (*N*=7), obesity (*N*=7), neuromuscular and central nervous system disorders (*N*=11), lower extremity and skin deficits (*N*=8), and cognitive impairment (*N*=5). Special populations included middle age/older adults (age > 42, *N*=1), suspected sepsis (*N*=2), and postsurgical (*N*=3).

**Figure 3:**
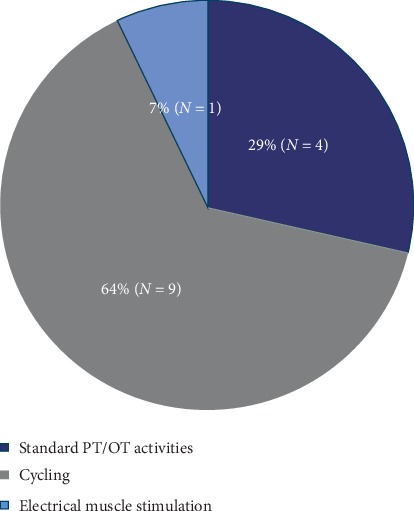
Primary early mobility intervention type studied. Abbreviations: PT, physiotherapy; OT, occupational therapy.

The primary EM intervention was standard physiotherapy using active and passive activity (*N*=4), cycling (*N*=9), and electrical muscle stimulation (*N*=1) (Figure 3). The EM protocols and the comparator group activities are described in Table S1. Studies utilizing cycling also offered regular physiotherapy sessions to patients, and 3 studies offered both cycling and electrical stimulation. Other components of the intervention were electrical stimulation either in combination with cycling or alone (*N*=3) and cognitive components (*N*=3). EM interventions were led by physiotherapists (*N*=2) and occupational therapists (*N*=1) and co-led by nursing and physiotherapy (*N*=1), with no specified leader in 10 studies. EM initiation was within 48 hours of ICU admission (*N*=4), not specified (*N*=4), or required the patient to be medically cleared prior to the initiation of EM (*N*=6).

**Figure 4:**
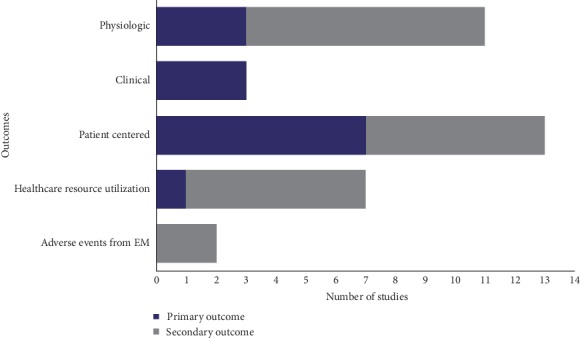
Primary and secondary study outcomes. Abbreviations: EM, early mobilization.

The primary outcome measures included physiological measures (*N*=3), clinical measures (*N*=3), patient-centered measures (*N*=7), and healthcare resource use (*N*=1; Figure 4). Patient-centered measures included functional status (*N*=2), quality of life (*N*=1), functional independence (*N*=1), and walking distance (*N*=3). Secondary outcomes measures included physiological measures (*N*=8), patient-centered measures (*N*=6), healthcare resource use (*N*=6), and adverse events (*N*=2). More than half of the studies (*N*=8/14) included a posthospitalization follow-up period. The most commonly used functional status measure was the 6-minute walk test (*N*=6). Other functional status measures used were the Physical Function ICU Test (*N*=3), the 4-item physical fitness in ICU test (*N*=1), Functional Status Score for the ICU (*N*=1), Short Physical Performance Battery (*N*=1), and Critical Care Physical Assessment Tool (*N*=1). Mobility was measured using the time up and go test (*N*=1) and the ICU mobility scale (*N*=1). Change in muscle mass and thickness was measured by the Medical Research Council Scale for Muscle Strength (*N*=2). Grip strength was measured using the Handheld Dynometry (*N*=3). Quality of life was measured with the Short Form 36 score (*N*=5) and EuroQol-5D-5L (*N*=2). Independence for activities of daily living was measured with the Barthel (*N*=4) and Lawton (*N*=1) scores. Cognition was measured with the Montreal Cognitive Assessment (*N*=3). Delirium was measured using the Confusion Assessment Method ICU score (*N*=4). Anxiety was measured with the Hospital Anxiety and Depression Scale (*N*=2). There were 2 studies that looked at inflammatory biomarkers at baseline and at discharge. ICU length of stay (*N*=4), hospital length of stay (*N*=2), number of days of mechanical ventilation (*N*=2), ventilator free days (*N*=1), and postoperative pulmonary complications (*N*=1) were analyzed. The safety of EM was also looked at by 2 studies by measuring rates of endotracheal tube dislodgement and central/arterial line dislodgement during activity. Posthospital follow-up was 1 month (*N*=1), between 2-3 months (*N*=3), 6 months (*N*=3), and 1 year (*N*=1).

## 4. Discussion

We identified 14 RCTs from two web-based clinical trial registries that are evaluating the impact of EM in an ICU setting. The majority of studies focused on patients who are undergoing or have been liberated from mechanical ventilation. Studies are small to medium-sized. Patients are being excluded from studies due to obesity, neuromuscular issues, and poor prognosis. There are no studies focused on older adults or acute cardiovascular disease patients. Cycling-based and electrical stimulation therapies are being studied, in addition to standard active and passive physiotherapy regimens. Outcome measures collected are heterogeneous. Most studies included posthospitalization follow-up.

Prior EM RCTs have focused on critically ill patients receiving mechanical ventilation in a general, medical, or cardiac surgical ICU [1, 10]. The patients included in these RCTs were typically adults with a mean age in the late 50 s. EM interventions included passive and active range of motion, mobility activities in and around the bed, upper limb exercises, and ambulation. There were methodological issues with prior studies, such as poor description of intervention arms, high rates of participant dropout, and lack of blinding of outcome assessors. A variety of functional outcome measures were used in prior studies, which precluded meta-analysis to characterize the impact of EM on functional outcomes. A Cochrane review of EM studies that included four RCTs reported studies using the Barthel Index, a measure of independent functional status, and the Acute Care Index of Function, the Physical Function ICU test score, and the Short Physical Performance Battery, as measures of physical performance [10]. Our study similarly found that upcoming RCTs are using a number of different functional measurement scales. There is a need to standardize functional measurement tools for reporting the effect of mobility interventions on functional status in the ICU.

Previous RCTs focused on standard physiotherapy-based protocols [1, 10]. Cycling-based activities and electrical stimulation, in addition to or as a replacement of standard physiotherapy, have emerged as therapeutic techniques for EM interventions. Cycling-based activities generally started with passive cycling with flexion/extension of lower extremities at a specific rate (i.e., # cycles/minute) for a predetermined duration with gradual increase in activity duration and intensity as tolerated. The advantages of cycling-based activities include the ability to deliver EM to patients who are mechanically ventilated and sedated as passive cycling. However, the patient is generally required to be hemodynamically stable prior to initiating cycling activities and patient participation is necessary when being advanced to active cycling. Electrical stimulation-based activities require electrodes to be attached to certain muscle groups such as hamstrings, quadriceps, and calves such that electrical impulses can be delivered to stimulate the muscle groups, which can be seen as visible contractions. The key advantages of electrical muscle stimulation are that it is an external stimulus, which does not require any participation from the patient and makes it feasible to be delivered in a critical care setting and can be delivered to patients without causing any hemodynamic instability. Prior studies investigating the impact of both cycling and EMS on muscle weakness and function in the ICU have had mixed findings. Both techniques are safe with low adverse event rates, but their impact on muscle wasting and strength have been mixed [12, 13]. A recent randomized trial in over 300 critically ill patients combined early in-bed cycling and EMS and did not find an improvement in muscle strength [14].

ICU survivors have been shown to suffer from postintensive care syndrome, which includes physical, psychological, and cognitive impairments leading to functional decline [15]. Functional outcomes, a key patient-centered measure, were frequently included as primary and/or secondary outcomes in the included studies. There is increasing recognition that many patients prioritize individual quality of life and functional independence over other more conventional societal measures [16]. EM may also improve mental health outcomes. An RCT looking at physiotherapy-led EM in mechanically ventilated patients found a trend toward improved mental health outcomes [17]. Several upcoming studies included cognitive components in addition to physical therapy, such as providing positive feedback to patients, performing cognitive tasks, or following strict checklists to allow spontaneous awakening trials and to address pain and delirium [18, 19]. A pilot RCT that combined cognitive therapy and early physical therapy with usual care in ICU patients with respiratory failure or shock did not show improvement in the intervention arm [18].

The effect of EM in other important critical care patient populations needs to be considered in future research design (Table 2). There are knowledge gaps in understanding the impact of EM, such as the timing of initiation of EM and the type and method of EM delivery. There are also certain populations that may stand to benefit from EM but have not been adequately explored, such as older adults, people with acute cardiovascular disease, people with preexisting mobility impairment, and nonmechanically ventilated critically ill patients [20]. Older adults may be more likely to benefit from an intervention that impacts functional status due to the higher burden of preexisting functional impairments and increased risk of functional decline during hospitalization. Prior EM studies largely excluded older adults and focused on younger overall cohorts [1, 21]. The absolute number and percentage of older adults in the ICU is growing as well [22]. There are also few studies evaluating EM in patients with acute CV disease [23]. In addition, patients with underlying neuromuscular/cerebral pathologies were excluded due to anticipated difficulty in weaning. These patients may still benefit from EM, as they are starting off at a disadvantage and therefore would be important to minimize further set-backs. To evaluate the impact of EM in the ICU on key outcome measures, there is a need for collaborative groups to obtain funding support to conduct large, multicenter trials to obtain high-quality data. However, achieving recruitment of large study population may be challenging in the ICU setting. We found two proposed studies with recruitment targets of over 700 patients. Previous RCTs in the ICU have shown considerable difficulty at meeting their target sample sizes for the primary outcome and many had to be stopped early due to insufficient accrual or logistical issues [24]. Strategies to boost study recruitment and to deal with potential logistical challenges, such as multicenter enrolment and the use of existing critical care trial research networks, should be considered in trial design.

Our study has a number of limitations. First, we used only two web-based clinical trial registries. There are other clinical trial registries available. Clinicaltrials.org is an international registry and contains the largest number of trial listings. In addition, a preliminary web and literature search indicated that there were potential studies to include in the ANZCTR registry and there were few potential studies in other trial registries. Second, we excluded certain ICU settings, such as poststroke and orthopedics, where a specialized approach to rehabilitation is required. Third, we attempted to contact the author to ensure that there was intent to publish the completed but unpublished studies. However, it is possible that some of these studies might not be published. One possible barrier to publish may have been difficulty establishing adequate follow-up. We also excluded studies that were completed over 5 years ago but were not yet published.

## 5. Conclusion

EM in the ICU has the potential to improve outcomes but current data on its' effectiveness are limited. The studies identified can yield more information regarding which EM modalities may be feasible, as well as the potential short- and long-term benefits. This review has identified areas that can be further explored, such as looking at specific study populations and looking at more innovative EM delivery methods.

## Figures and Tables

**Table 1:** Ongoing and unpublished randomized trials of early mobilization interventions in the ICU on Clinicaltrials.gov.

Study identifier	Title	PI/country	Inclusion criteria	N	Study arms	Outcomes	Status, completion date
NCT02520193	Impact of Early Mobilization on Mechanical Ventilation Duration in Intubated Critically Ill Patients (EarlyMob)	Poiroux/Belgium	Age ≥ 18 Admission to ICU MV for 24–48 hours	772	I: protocolized EM strategy C: standard mobilization strategy	(1) Number of days without MV (2) Incidence of ICUAW, pressure ulcers, delirium, hospital LOS, place of transfer at discharge, days between intubation to spontaneous breathing trial, extubation failure.Time from MV-stand up, ICU length of stay	Unknown 2018

NCT02872792	Early Mobilization in Intensive Care Unit: Interest of Cyclo-Ergometry in Patients with Septic Chock (MUEVELO)	Beduneau/ France	Age ≥ 18 Hospitalized in ICU Septic shock dx ≥ 24 hours HD stable before 72 hours of MV, RASS ≤ −2 BMI ≤ 40 Contraception (WOCBA)	234	I: EM with cyclo-ergometer daily in addition to standard PT C: standard PT	(1) Number of days between HD stability and discharge from ICU (2) Number of days between HD stability and cessation of sedation (3) Number of days under MV	Recruiting 2019

NCT02300662	Early Mobilization for Critical Patients on Invasive Mechanical Ventilation in the Intensive Care Unit (MoVe-ICU)	Vierira/ Brazil	Age ≥ 18 ICU transfer ≤ 1 week after ER/ward MV 24–48 hours	28	I: conventional PT and cycle ergometer for 20 minutes, passive movement of cycle—flexion/extension of knees and hips C: conventional PT	(1) Change in cross-sectional quadriceps thickness (2) Change in length of fascicle, pennation angle, thickness of vastus lateralis, diaphragm thickness and excursion	Completed 2015

NCT02312648	Impact of Mobilization on Cardiac Surgery	Chiavegato/Brazil	Age 21–90 Elective CABG BMI 20–30 MV < 24 hours HD stable ± pressors No arrhythmia/angina MAP 60–100 HR 60–100 No respiratory distress	52	I: deep breath until POD#7, NIV for 30–60 minutes post extubation Upper + lower limb ergometer exercises, limb exercises, chair transfer C: respiratory exercises, NIV for 30–60 minutes post-extubation	(1) Functional capacity—preoperative, at hospital discharge, and 60 days postoperative (6-minute walk test) (2) ICU and hospital length of stay, postoperative pulmonary complication	Completed 2016

NCT02864745	Early Mobilization and Intensive Rehabilitation in the Critically Ill (EMIR)	Duska/Czech Republic	Age ≥ 18 MV or imminent need Predicted ICU LOS ≥7 days	150	I: very early (<48 hours), protocolized, intensive rehabilitation—functional electrical stimulation-assisted cycle ergometry C: standard rehab delivered by non-study PT	(1) Quality of life—Short Form 36 score (2) 4-item physical fitness in ICU, muscle mass, nitrogen balance, muscle power, number ventilator free days, number rehab interruptions, number episodes of elevated ICP, number dialysis interruptions, ICU LOS	Recruiting 2019

NCT03554811	Early Rehabilitation using Functional Electrical Stimulation-Assisted Supine Cycling in the Intensive Care Unit	Smith/USA	Age ≥ 18 Predicted ICU LOS ≥4 days Expected ICU survival Expected MV > 48 hours Can perform outcome measures premorbidly	32	I: functional electrical stimulation-assisted supine cycling within 48 hours of ICU admission C: conventional early exercise and mobility interventions—standard ICU exercise and mobility as per alertness and stability	(1) Percent change of rectus femoris cross-sectional area (baseline, weekly, at ICU discharge and hospital discharge) (2) Diaphragm muscle thickness by ultrasound, muscle strength (MRCS), physical function (PFIT, FFS-ICU, SPPB, 6MWT), quality of life (SF-36 survey), cognition (MoCA), hospital LOS, ICU LOS, duration of MV	Not yet recruiting 2020

NCT03229070	Cycle Ergometer in the Postoperative of Thoracic Surgery (CE_PTS)	Macagna/ Brazil	Age 30–80 Planned thoracotomy, VATS for pulmonary resection Extubated HD stable MAP 60–100, HR 50–100 O_2_ saturation ≥ 90% Prescription for respiratory and motor PT	135	IA: interval effort group: high load -> active recovery phase with light to moderate load 1B: continuous effort group: mild to moderate intensity C: standard care	(1) 6MWT (2) Number of success on lift and sit on chair	Not yet recruiting 2018

NCT03133377	Treatment of Invasively Ventilated Adults with Early Activity and Mobilization (TEAM (III))	Hodgson/Australia	Age ≥ 18 MV for 2 or more days HD stable Respiratory stability	750	I: daily assessment by ICU PT using IMS scale, protocol is hierarchical C: standard care by PT	(1) Number of days alive and out of hospital (2) Mortality, ventilator-free days, ICU-free days, quality of life, EQ5D-5L, Barthel's basic activities of daily living, Lawton's instrumental activities of daily living, WHODAS. Others: delirium-free days, MoCA, HADS, and IES-R scores	Recruiting 2021

NCT03771014	A Feasibility Study of Early Mobilization Programmes in Critical Care (EMPRESS)	Cusack/United Kingdom	Age ≥ 42 ICU admission Independent (barthel > 80) in hospital <5 days Expected MV for 48 hours	90	I: standard PT regimen and 2 times 30 minutes rehabilitation sessions 5 days per week C: standard care PT	(1) Physical function ICU test score (2) Muscle strength and function MRC, handheld dynamometry, CPAX, time up and go, clinical frailty score, Barthel ADL, 6MWT, HADS WHODAS, EQ5D-5L	Not yet recruiting 2021

NCT03770442	Muscle Wasting in the Critically Ill	Welters/United Kingdom	Age 18–90 ICU admission MV and initial sedation Definite or suspected sepsis of any source	36	I: cycling with functional electrical stimulation (FES). Will also receive routine PT. C: routine PT	(1) Change in muscle thickness or fascicle pennation angle of various muscles. Change in thickness with respiration. (2) Change in blood or urine biomarkers, 6MWT, hand grip dynamometry, limb strength, balance, SF-36, MIP, CAM ICU, RRT, total dose of noradrenaline, fluid balance, insulin per day, glucose, HR variability, safety measures	Not yet recruiting 2020

NCT01705015	Organ Transplantation Rehabilitation: Effect of Bedside Exercise Devices and Activity Reinforcement	Chen/Taiwan	Age 18–80 After heart or liver transplant at NTUH Independent 6 weeks preadmission	110	I: ICFit and direct feedback—encouraged to look at summary of daily data and encouraged to pedal C: UCFit and encouraged—graph of mins cycled left at bedside	(1) Level of independence for walking 150 feet; walking speed for 50 feet; 6MWT; cardiopulmonary exercise testing (2) total exercise; SF-36; LOS; rate of rehospitalization; complications to exercise	Unknown 2015

ACTRN12618000374268	The Effectiveness of Early Functional Occupation-Based Retraining Therapy in a Medical/Surgical Intensive Care Unit	Rapolthy-Beck/Australia	Age ≥ 18 ICU admission Expected MV ≥ 48 hours	30	I: (I) performance-based and cognitive stimulation (II) Qualitative component –explore experience of participants C: usual OT care	(1) Independence for activities of daily living (FIM) at discharge and 90 days follow-up (2) Functional ability (modified Barthel's), MoCA, dynamometer, SF-36, HADS, RASS, GCS, CAM-ICU	Recruiting 2020

ACTRN12614001059651	Effects of Combined Electrical Muscle Stimulation and Resistance Exercises in Duration of Mechanical Ventilation in Critically Ill Patients	Vieira/ Brazil	Age ≥ 18 ICU admission MV	40	I: resistance exercise, electrical mechanical stimulation, or both C: usual care by respiratory PT	(1) Duration of MV (2) LOS in ICU, biomarker analysis (IGF-1, IL1B, IL-6, IL-10, IL-18, TNF-a) performed at baseline and at ICU discharge	Completed 2015

ACTRN12614000763640	A Randomized Controlled Study of the Awakening and Breathing Trial Coordination; Delirium Monitoring and Management; and Early Exercise and Mobility (ABCDE) Bundle to Improve Functional and Cognitive Capacity in Ventilated Critically Ill Patients	Sosnowski/Australia	Age 18–99 MV ≥ 48 hours	100	I: daily SBT and SAT, delirium assessment and exercise to identify etiology and minimize, target RASS C: standard therapy without use of any protocols	(1) Physical function ICU test, MoCA, and Barthel index (2) RASS scale, CAM-ICU	Not yet recruiting 2014

Abbreviations: 6MWT, 6-minute walk test; Barthel's ADL, Barthel's activity of daily living; BMI, body mass index; CABG, coronary artery bypass graft; CAM ICU, confusion assessment method for the ICU; CPAX, Chelsea critical care physical assessment tool; Dx, diagnosed; GCS, Glasgow Coma Scale; HD, hemodynamic; EQ5D-5L; 5 Level EuroQol 5 dimension; ER, emergency room; FFS-ICU, Functional Status Score for the Intensive Care Unit; FIM, functional independence measure; HADS, Hospital Anxiety and Depression Scale; HR, heart rate; ICP, intracranial pressure; ICU, intensive care unit; ICUAW, intensive care unit acquired weakness; IES-R, Impact of Event Scale-Revised; IGF-1, insulin-like growth factor 1; IL1b, interleukin 1b; IL6, interleukin 6; IL10, interleukin 10; IL18, interleukin 18; IMS, ICU Mobility Scale; LOS, length of stay; MAP, mean arterial pressure; MIP, maximal inspiratory pressure; MoCA, Montreal Cognitive Assessment; MRCS, Medical Research Council Scale; MV, mechanical ventilation; NIV, noninvasive ventilation; OT, occupational therapy; PFIT, Physical Function ICU Test; POD, postop day; PT, physiotherapy; RASS, Richmond Agitation Sedation Scale; RRT, renal replacement therapy; SAT, spontaneous awakening trial; SBT, spontaneous breathing trial; SF-36; Short Form 36; SPPB, Short Physical Performance Battery; TNFa, tumor necrosis factor alpha; QoL, quality of life; VATS, video-assisted thoracoscopic surgery; WOCBA, woman of child bearing age; O2, oxygen; WHODAS, World Health Organization Disability Assessment Schedule.

**Table 2:** Limitations and future directions for early mobility research.

Limitations of current evidence base	Study populations that should be explored	Knowledge gaps
Mechanically ventilated patients	Older adults	Timing for initiation of mechanical ventilation
Primary respiratory disease	Nonmechanically ventilated critically ill patients	Nurse-led early mobilization
Few RCTs with heterogeneous outcome measures	Acute cardiovascular patients	Patient-centered outcomes
No evidence of functional benefit	Preexisting mobility impairment	Standardized protocol for EM (i.e., nature of activity, personnel of delivery)
No large RCTs	Neuromuscular impairments	
	Obese patients	
	Patients with cognitive impairment	
	Gender differences	

Abbreviations: RCT, randomized controlled trials; EM, early mobilization.

## Data Availability

The data used to support the findings of this study are included within the article, under the figure and table sections.

## Supplementary Materials

Supplementary Materials Supplementary Table 1 (S1): a detailed description of the intervention and comparison for each clinical trial. Details of the nature of early mobilization are described, such as the type of activity and timing of initiation and the duration and frequency of the intervention, if details were provided.
