# Supplementary material for: Early Mobilization Interventions in the Intensive Care Unit: Ongoing and Unpublished Randomized Trials
Source: Crit Care Res Pract. 2020 Jan 21;2020:3281394. doi: 10.1155/2020/3281394 (PMC7201471; doi:10.1155/2020/3281394)
Supplement: Supplementary Materials — Supplementary Table 1 (S1): a detailed description of the intervention and comparison for each clinical trial. Details of the nature of early mobilization are described, such as the type of activity and timing of initiation and the duration and frequency of the intervention, if details were provided. [file 3281394.f1.docx]

**Table S1: Details of early mobilization protocol and comparison of included studies.**

| **Study** | **Early Mobilization** | **Comparison** |
| --- | --- | --- |
| Early Mobilisation in Intensive Care Unit : Interest of Cyclo-ergometry in Patients With Septic Chock (MUEVELO)  NCT02872792 | Daily early mobilization with cycle ergometer provided, in addition to standard physiotherapy.  No further details provided. | Standard physiotherapy: Passive and active mobilization (sitting on bed/chair, walking) |
| Early Mobilization for Critical Patients on Invasive Mechanical Ventilation in the Intensive Care Unit (MoVe-ICU)  NCT02300662 | Cycle ergometer at 20 cycles/min for 20 minutes. Flexion and extension of lower extremity muscle groups required. Activity done daily while on mechanical ventilation. Standard physiotherapy with respiratory care also provided. | Standard physiotherapy with respiratory care for optimization of secretion clearance and vibrocompressions. |
| Impact of Mobilization on Cardiac Surgery  NCT02312648 | Active cycle ergometer of limbs for 20 minutes including a heating period of 5 minutes, low intensity exercise at 30 rpm for 10 minutes, and a 5 minute recovery period. Standard respiratory exercise also done. Activity done until post op day 7. | Standard respiratory exercise where sitting position is maintained and breathing exercises done - 3 sets of 10 repetitions of deep breathing. |
| Early Mobilization and Intensive Rehabilitation in the Critically Ill (EMIR)  NCT02864745 | Cycle ergometer assisted by electrical muscle stimulation initiated within 48 hours of ICU admission. Protocol not available. | Standard rehabilitation provided by physiotherapist. No further details provided. |
| Early Rehabilitation Using Functional Electrical Stimulation Assisted Supine Cycling in the Intensive Care Unit  NCT03554811 | Supine cycle ergometer assisted by functional electrical stimulation provided with surface electrodes applied to hamstrings, quads, and calf muscles. Each cycle involve 1 minute of passive cycling warm up at 20 rpm. Active and passive cycling according to participation is continued. Intervention done within 48 hours of admission and provided for up to 1 hour per day, Conv5 days per week for 28 days or until discharge from ICU. | Conventional early exercise and mobility interventions based on medical stability. Activities done to increase range of motion and strength of muscle groups and progressive ambulation. |
| Cycle-ergometer in the Postoperative of Thoracic Surgery (CE_PTS)  NCT03229070 | Interval effort group: Cycle ergometer with high load lasting 60 seconds (high load active phase) with target pedaling speed of 30-60rpm for 5 cycles, lasting 20 minutes. then active recovery phase for 4 minutes. Continuous effort group: 20 minutes of mild to moderate intensity pedaling speeds between 30-60rpm. | No intervention. |
| A feasibility Study of Early Mobilisation Programmes in Critical Care (EMPRESS)  NCT03771014 | Passive cycling with progression with assisted cycling, active cycling, exercises in bed and mobilization. Started within 48 hours and provided 30 minutes x 2 sessions per week, 5 days per week. | Standard physiotherapy. No further details available. |
| Muscle wasting in the critically ill  NCT03770442 | Functional electrical stimulation assisted cycle ergometry with stimulation to specific lower extremity muscle groups and abdomen. Started within 48 hours of ICU admission, 30 minutes each session with a total of 10 sessions of 14 days. | Standard physiotherapy with respiratory care. No further details available. |
| Organ Transplantation rehabilitation: Effect of bedside exercise devices and activity reinforcement  NCT01705015 | Positive patient feedback given with encouragement to progress with activity level by providing patient their summary of daily activity, which is left at the bedside. Patients are encouraged to increase reps from 10-60rpm, have more frequent sessions up to 3 times/day, do longer sessions from 5-20 minutes, and encouraged to exert against greater forces. Once daily pedaling of upper extremities and progression of lower extremity activity was encouraged. | Patients provided with graph of their activity, however, no further positive feedback given. |
| Effects of combined electrical muscle stimulation and resistance exercises in duration of mechanical ventilation in critically ill patients  ACTRN12614001059651 | Resistance exercise group: passive mobilization then resistance with elastic band for active exercises. Done once daily until discharged from ICU.  Electrical muscle stimulation group: electrical muscle stimulation provided by stimulators on various lower extremity muscle groups (vastus lateralis and medialis) with biphasic impulses of 45Hz of 400 microseconds pulse for 12 seconds on and 6 seconds off. Intensities are required to either cause visible contractions or palpable contractions. Sessions last 55 minutes total including a 5 minute warm-up and 5 minute recovery time.  Combined resistance exercise and electrical muscle stimulation: Both techniques provided at the same time.  Twice daily respiratory physiotherapy of 30 minutes provided to all patients. | Twice daily respiratory therapy with positioning and mucous clearing techniques, 30 minutes per session, provided. |
| Impact of Early Mobilization on Mechanical Ventilation Duration in Intubated Critically Ill Patients (EarlyMob)  NCT02520193 | Protocolized early mobilization. No further details provided. | No details on comparison. |
| Treatment of Invasively Ventilated Adults With Early Activity and Mobilisation (TEAM(III))  NCT03133377 | Daily physiotherapy led early mobilization done using hierarchical early activity and mobilization protocol. ICU mobility scale helped guide nature and frequency of activity. Activities typically started at highest level of activity tolerated for longest time and intensity decreased if patient fatigues. | Standard care by physiotherapist. No further details provided. |
| The effectiveness of early functional occupation-based retraining therapy in a medical / surgical intensive care unit.  ACTRN12618000374268 | Occupational therapy led early purposeful activity done daily for maximum 60 minutes. Activities based on pre-hospitalization lifestyle. Cognitive stimulation tasks provided according to cognitive level. Sensory stimulation and modulation done with orientation and reflection. Functional tasks included grooming related tasks, self care activities in bed to bathroom, and leisure related activities. | Standard care by occupational therapist. No further details provided. |
| A randomized controlled study of the Awakening and Breathing trial Coordination; Delirium monitoring and management; and Early exercise and mobility (ABCDE) bundle to improve functional and cognitive capacity in ventilated critically ill patients.  ACTRN12614000763640 | Protocolized ICU care with daily awakening, breathing trials, monitoring of delirium with active efforts at identifying etiology. Once deemed safe for early mobilization, graded level of activities done, starting with level 1: passive range of motion 3x/day, active resistance physiotherapy 2x/day, and sitting position in bed 3x/day for 20 minutes each. Level 4: sitting on edge of bed, active transfer to chair and ambulation daily. | Standard care with no use of protocols. Daily vs. twice daily exercise activity provided by physiotherapist, as deemed appropriate. |

**Supplementary Table 1 (S1):** A detailed description of the intervention and comparison for each clinical trial. Details of the nature of early mobilization are described, such as the type of activity and timing of initiation, the duration and frequency of the intervention, if details were provided.
